# Spontaneous ischaemic stroke lesions in a dog brain: neuropathological characterisation and comparison to human ischaemic stroke

**DOI:** 10.1186/s13028-016-0275-7

**Published:** 2017-01-13

**Authors:** Barbara Blicher Thomsen, Hanne Gredal, Martin Wirenfeldt, Bjarne Winther Kristensen, Bettina Hjelm Clausen, Anders Elm Larsen, Bente Finsen, Mette Berendt, Kate Lykke Lambertsen

**Affiliations:** 1 Department of Veterinary Clinical Sciences, Faculty of Health and Medical Sciences, University of Copenhagen, 1870 Frederiksberg, Denmark; 2 Department of Pathology, Odense University Hospital, 5000 Odense, Denmark; 3 Department of Neurobiology Research, Institute of Molecular Medicine, University of Southern Denmark, 5000 Odense, Denmark; 4 Department of Neurology, Odense University Hospital, 5000 Odense C, Denmark; 5 BRIDGE, Brain Research – Inter-Disciplinary Guided Excellence, Department of Clinical Research, University of Southern Denmark, 5000 Odense, Denmark

**Keywords:** Animal model, Astrocyte, Canine, Cerebral infarction, Cerebrovascular accident, Infarct, Microglia, Middle cerebral artery occlusion

## Abstract

**Background:**

Dogs develop spontaneous ischaemic stroke with a clinical picture closely resembling human ischaemic stroke patients. Animal stroke models have been developed, but it has proved difficult to translate results obtained from such models into successful therapeutic strategies in human stroke patients. In order to face this apparent translational gap within stroke research, dogs with ischaemic stroke constitute an opportunity to study the neuropathology of ischaemic stroke in an animal species.

**Case presentation:**

A 7 years and 8 months old female neutered Rottweiler dog suffered a middle cerebral artery infarct and was euthanized 3 days after onset of neurological signs. The brain was subjected to histopathology and immunohistochemistry. Neuropathological changes were characterised by a pan-necrotic infarct surrounded by peri-infarct injured neurons and reactive microglia/macrophages and astrocytes.

**Conclusions:**

The neuropathological changes reported in the present study were similar to findings in human patients with ischaemic stroke. The dog with spontaneous ischaemic stroke is of interest as a complementary spontaneous animal model for further neuropathological studies.

## Background

Dogs suffer from spontaneous ischaemic stroke with neurological signs and magnetic resonance imaging (MRI) findings largely comparable to those of humans [1, 2]. Like humans, dogs with ischaemic stroke display variable neurological signs depending on the topography of the vascular occlusion and the size of the infarct [1, 3–5].

Experimental rodent models have provided extensive knowledge of the pathophysiological mechanisms of ischaemic stroke [6, 7]. It has, however, proved difficult to translate results obtained from such models into successful therapeutic strategies in human stroke patients [8, 9]. In order to face this apparent translational gap within stroke research, it has been proposed to search for alternative animal models comprising more aspects of the human disease [10].

Dogs resemble humans with regard to basic anatomy of a large-sized gyrencephalic brain, its vascularization and a high ratio of white compared to grey matter [11–13]. Furthermore, dogs age naturally and, as humans, they experience diseases of longevity such as cardiovascular disease and diabetes mellitus. They are also exposed to similar risk factors for ischaemic stroke, including obesity, hypertension and environmental exposures such as pollution and passive smoking.

Histopathological reports of ischaemic stroke in dogs are sparse and studies including a detailed evaluation of morphological changes of neurons, microglia/macrophages, and astrocytes in combination are still lacking [14–28]. In humans, neuroglia are recognized as central components of the pathophysiology of ischaemic stroke, and especially microglia have in recent years gained attention in basic research, as these cells can exert both beneficial and detrimental effects on neurons situated in peri-infarct lesions [7, 29–32].

The aim of the present study was to report histopathological findings with an emphasis on neuroglial reactions in the infarct and adjacent (peri-infarct) areas in a canine brain with a spontaneously occurring middle cerebral artery (MCA) infarct. The translational potential of canine ischaemic stroke as a spontaneous animal model of human ischaemic stroke is discussed.

## Case presentation

**Fig. 1:**
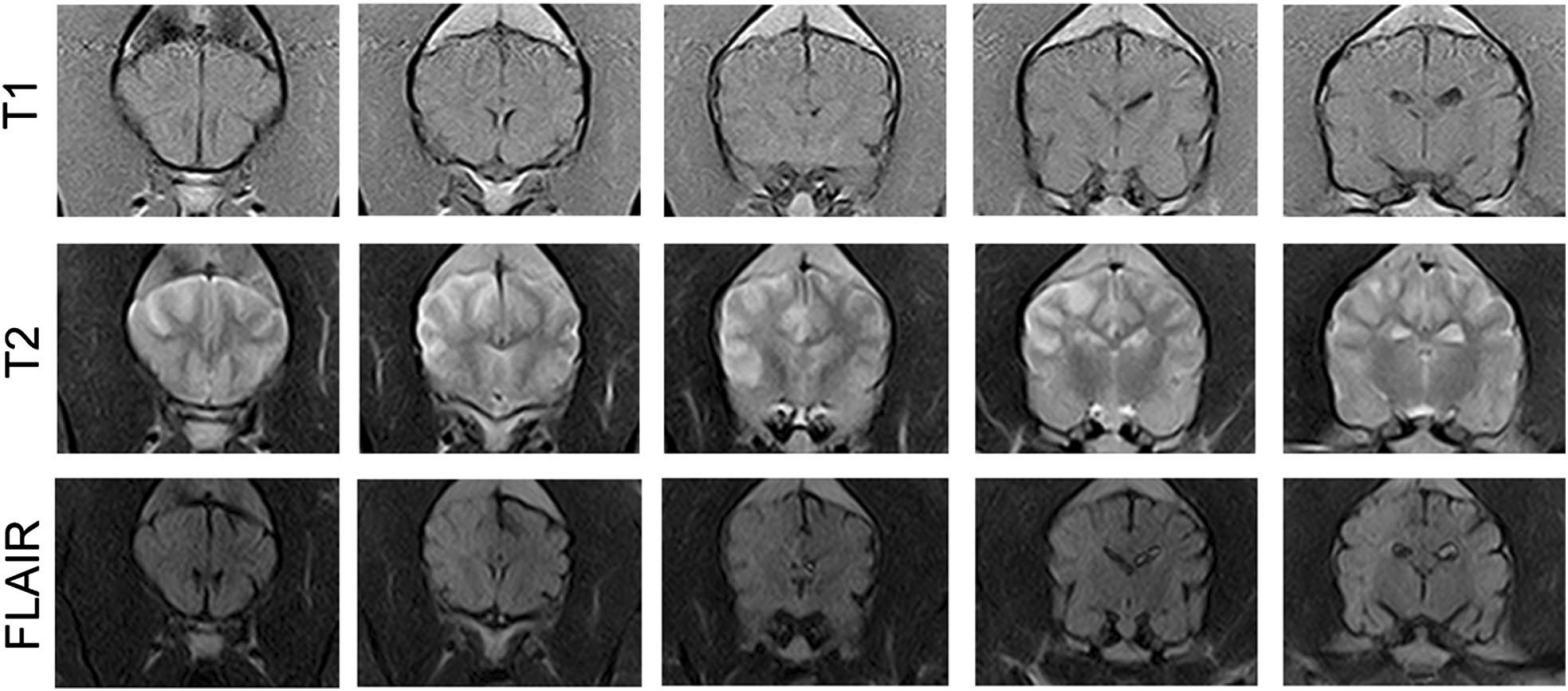
Magnetic resonance imaging of stroke-lesioned canine brain. Sequential magnetic resonance images of coronal sections at the level of the parietal and temporal lobe from a dog performed 2 days after onset of the ischaemic stroke. Direction of images: rostral to caudal. Images were obtained with a 0.2 T MRI (Vet-MR, Esaote). *Upper row* No signal changes are seen in T1 images. *Middle row* Hyperintense signals are seen in T2. *Lower row* Hyperintense signals are seen in FLAIR. Hyperintensity is reflecting parenchymal changes following the ischaemic infarct

A 7 years and 8 months old female neutered Rottweiler dog presented at the University Hospital for Companion Animals, University of Copenhagen, Denmark with an acute onset of left-sided non-ambulatory hemiparesis and left-sided hemineglect. Otherwise, physical examination was unremarkable and complete blood count, biochemistry, thromboelastography, urinalysis, and cerebrospinal fluid analysis were normal. The dog had low levels of thyroxin (T4): ≪ 6.44 nmol/l (11.2–40.8) and free T4: ≪ 3.86 pmol/l (7.7–47.6) and increased levels of thyroid stimulating hormone (TSH): 0.62 ng/ml (0.00–0.50) but showed no clinical signs of hypothyroidism. MRI findings were compatible with a spontaneous right-sided MCA occlusion (Fig. 1). The dog was euthanized 3 days after initial presentation at the owners’ request, and the brain was donated for post-mortem studies.

The brain of a 1 year and 7 months old healthy female mixed-breed dog, euthanized at the owner’s request and donated to the University Hospital for Companion Animals for teaching and research purposes, was used as a normal control for the development of immunohistochemical (IHC) protocols and for control sections.

The use of the canine tissues was approved by the Local Administrative and Ethics Committee, Department of Veterinary Clinical and Animal Sciences, Faculty of Health and Medical Sciences, University of Copenhagen (Permission number 1 N/2013).

### Processing of brain tissue

The brain of the ischaemic stroke case was collected within 2 h post-mortem. The brain was fixed by immersion in 4% formaldehyde for 14 weeks and stored in 0.15 M phosphate-buffered saline (PBS) with 30% sucrose and 0.1% sodium azide (pH 7.4) for 15 months at 4 °C. The brain was cut transversally into 19 slabs of 5 mm thickness. Each individual brain slab was numbered and photographed with the rostral cut-surface pointing upwards. Meninges were removed and the slabs were divided into smaller pieces in order to fit the vibratome equipment. Each piece was embedded in agar and further divided into 24 series of 70-μm thick, free-floating sections on a vibratome (Leica VT1000 S, Leica Microsystems, Ballerup, Denmark). Sections were stored in de Olmos cryoprotectant solution containing polyvinylpyrrolidone and sucrose diluted in a mixture of ethylene glycol and PBS and stored at −12 °C until further processing.

### Histochemical staining

Every 24th section was stained with a solution of 0.01% toluidine blue (TB) (Merck Millipore, Hellerup, Denmark) diluted in 0.08 M Na_2_HPO_4_∙2H_2_O and 0.07 M citric acid in distilled H_2_O [34], and a luxol fast blue (LFB) solution (Amplicon, Odense, Denmark) [35], respectively. Sections were rinsed overnight in tris-buffered saline (TBS) at pH 7.4 and then mounted on gelatine-coated glass-slides and air-dried. For TB staining, slides were placed in TB for 26 min and differentiation was subsequently performed in graded series of alcohol and cleared in xylene. For LFB staining, differentiation of the sections was started by placing the sections in series of graded alcohol, and sections were then placed in LFB solution overnight at 4 **°**C. Next day, differentiation was continued by placing the sections in 0.05% lithiumcarbonate for 3 min and sections were counterstained using haematoxylin and eosin (HE). Coverslipping was performed using Depex mounting medium (VWR, Herlev, Denmark).

### Immunohistochemistry

Every 24th section of the free-floating sections was selected for IHC detection of microglial Iba1 and glial fibrillary acidic protein (GFAP) in astrocytes. Rinsing and incubation procedures were performed at room temperature, unless otherwise stated. Sections were rinsed 2 × 30 min in 0.05 M TBS, pH 7.4, and then left overnight in the same solution at 4 °C. Demasking was performed by rinsing sections 2 × 15 min in a tris-EGTA buffer (TEG) followed by heat induced epitope retrieval by heating sections in TEG in a microwave oven (Moulinex Optimo Duo, Groupe SEB, Ballerup, Denmark) for 2 × 4 min at 800 W and 1 × 10 min at 480 W or until boiling. Sections were then rinsed 30 min in TBS followed by 3 × 25 min in TBS + 1% Triton X, preincubated with 10% foetal calf serum (FCS) in TBS for 1 h and incubated for 3 days at 4 °C with one of the following primary antibodies: polyclonal rabbit anti-Iba1 (1:500, Wako-Chem, Osaka, Japan) or polyclonal rabbit anti-GFAP (1:200, Dako, Glostrup, Denmark) diluted in 10% FCS in TBS. Next, sections were rinsed 3 × 15 min in TBS, 30 min in TBS + 1% Triton-X, 15 min in TBS and blocked for endogenous peroxidase activity for 30 min in 100% methanol and 0.2% hydrogen peroxide. Rinsing was then performed 15 min in TBS and 2 × 60 min in TBS + 1% Triton-X. Sections were then incubated with EnVision™ + System-HRP (Dako) overnight at 4 °C. Next, all sections were rinsed 3 × 45 min in TBS and developed in 0.05% 3,3′-diaminobenzidine (DAB) and 0.033% hydrogen peroxide. Sections were then rinsed 2 × 30 min in TBS and 30 min in a tris-buffer. Finally, sections were mounted on gelatin-coated glass-slides, and when air-dried, counterstained with TB diluted in tris-buffer to a 3/4 solution for 16 min, dehydrated in graded alcohol, cleared in xylene and mounted with Depex (VWR).

Control for antibody specificity was performed on brain tissue sections of the control dog by substituting the primary antibody with rabbit IgG (Dako) and by omitting the primary antibody in the protocol. All sections were devoid of immunostaining.

### Neuropathological examination

Gross examination of the ischaemic stroke brain immediately upon removal from the skull revealed a soft and oedematous area with a diameter of approximately 20 mm, which was visible on the surface of the right cerebral hemisphere in the lateral communication of the frontal and parietal lobes. A detailed examination of the brain after fixation and sectioning into slabs revealed a swollen area protruding above the natural convex curve of the right frontal and parietal cerebral lobes with flattened gyri and narrowed sulci. This location corresponded to the affected area as visualized by MRI. The lesion measured, in medial–lateral direction up to 32 mm, in ventral-dorsal direction up to 36 mm, and in rostro-caudal direction up to 35 mm. The lesion involved neocortical grey matter and centrum semiovale white matter in the caudal part of the right frontal lobe, the right parietal lobe, the lateral and superior part of the right temporal lobe, and the most caudal part of the right hippocampus. The medial parts of the right frontal and parietal lobes towards the cerebral falx, including the cingulate gyrus, the medial parts of the superior frontal gyrus and the most medial aspects of the right temporal lobe, were spared. Likewise did the corpus callosum, basal nuclei, thalamus, brainstem, and cerebellum appear normal.

**Fig. 2:**
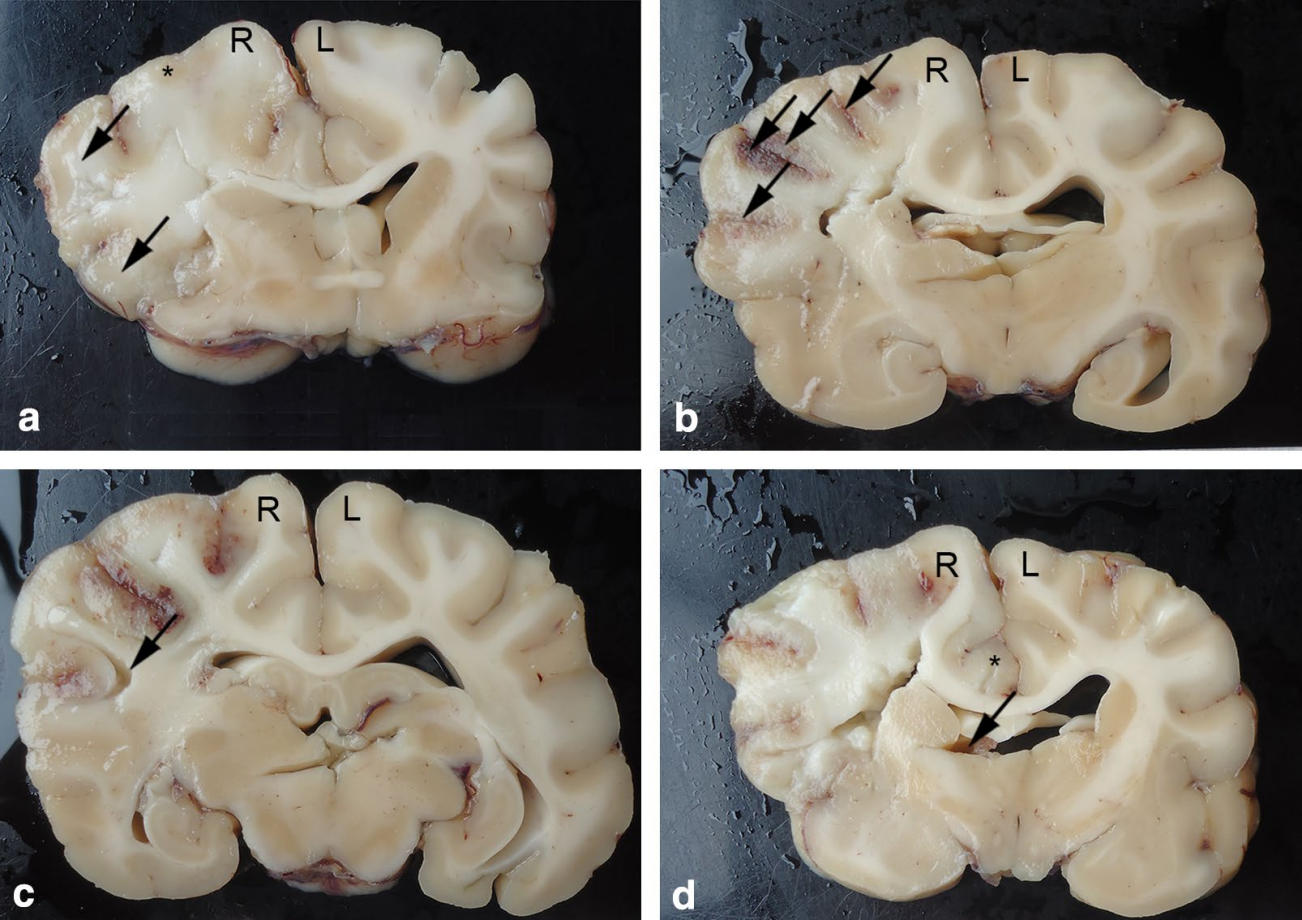
Gross lesions in the canine brain with a right-sided middle cerebral artery infarct. **a** Swollen and flattened gyri with narrowed sulci (*arrows*). Poor demarcation of grey/white matter interface and a dusky discoloration of the grey matter (*asterix*). Transverse section at the level of the basal nuclei. **b** Focal petechial haemorrhages in the grey matter of several sulci (*arrows*). Transverse section at the level of the thalamus. **c** Focal detachment of neocortex from underlying white matter (*arrows*). Transverse section at the level of the thalamus. **d** Narrowed and compressed right lateral ventricle (*arrow*). Subfalcine herniation with displacement of the right cingulate gyrus (*asterix*). Note the general grainy appearance of the neural tissue caused by oedema leading to asymmetry of the hemispheres and midline shift towards the left hemisphere. Transverse section at the level of the caudate nucleus. *R* right cerebral hemisphere. *L* left cerebral hemisphere

The lesion blurred the grey/white matter interface, caused a dusky discoloration of the grey matter, and had a cracking, friable appearance (Fig. 2a). A distinct boundary between the lesion and the surrounding brain parenchyma was evident. The lesion core was predominantly bland. However, focal petechial haemorrhages were present in the grey matter along several sulci (Fig. 2b) indicating haemorrhage from reperfusion of damaged vessels and tissue, typically associated with embolic events. The neocortex appeared focally detached from the underlying white matter in a circumscribed pattern of cortical laminar necrosis (Fig. 2c). Cerebral oedema was manifest with a grainy, coarse appearance of the cut surface in the suspected ischaemic area of the affected slabs. An obvious midline shift towards the unaffected left hemisphere and subfalcine herniation with displacement of the right cingulate gyrus under the falx cerebri and compression of the cavity of the right lateral ventricle was present (Fig. 2d). The pathological changes in the brain parenchyma corresponded to an ischaemic lesion caused by cessation of blood flow in the vascular territory of the right MCA. No blood vessel thrombus or embolus was identified.

**Fig. 3:**
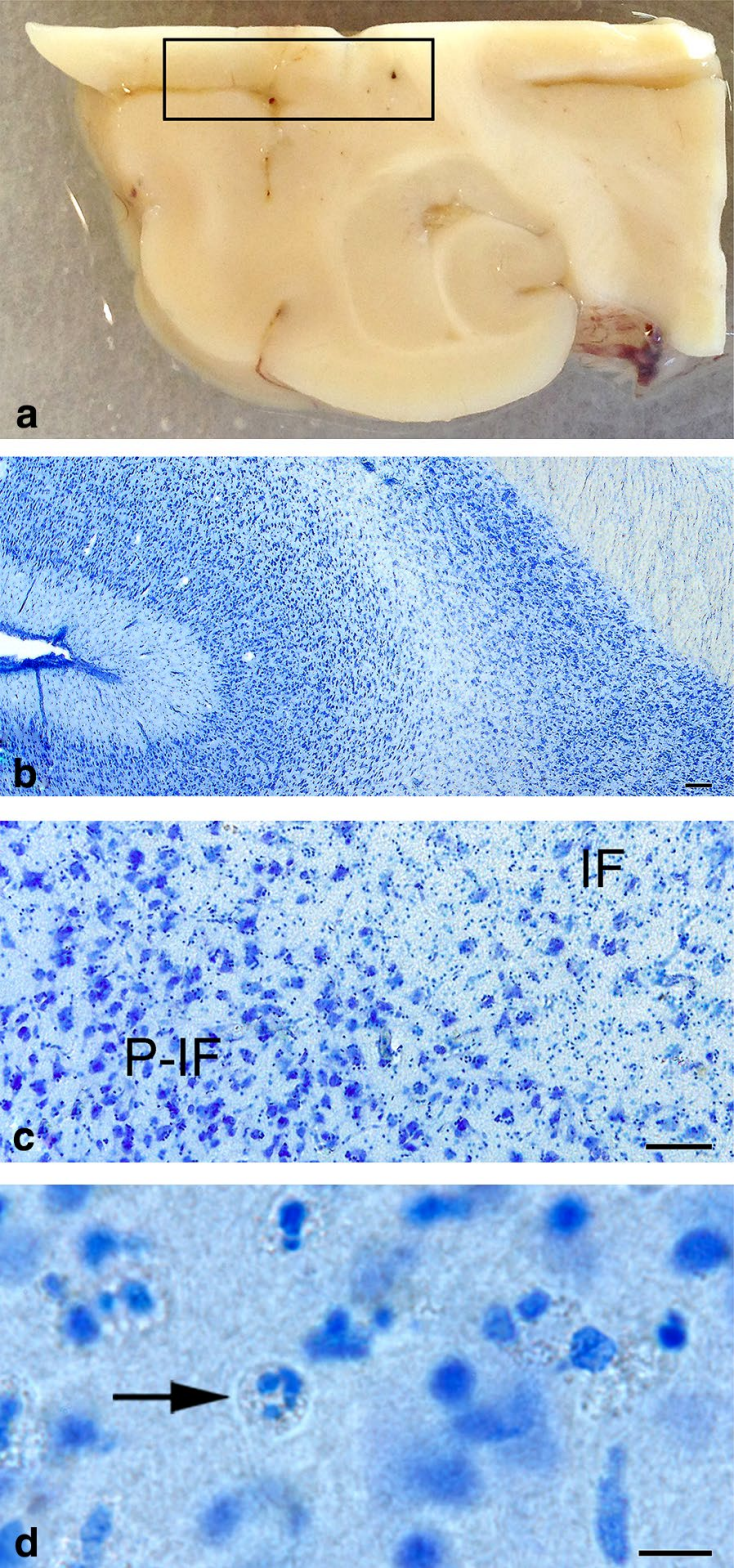
Topographic overview of canine brain tissue selected for histopathological evaluation. **a** Brain slab divided for vibratome processing. *Box* Area of the infarct and adjacent neuroparenchyma. **b**–**d** Tissue in *box* stained toluidine blue. *IF* infarct. *P*-*IF* peri-infarct area. *Arrow* Neutrophil granulocyte. *Bars* **b** = 200 μm, **c** = 100 μm, **d** = 10 μm

**Fig. 4:**
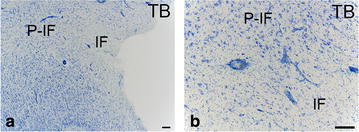
Photomicrographs showing the cortical peri-infarct zone in the dog brain. TB: toluidine blue. *IF* infarct. *P*-*IF* peri-infarct. *Bars* 200 μm. Note the loss of neurons and glial cells in the infarct area

**Fig. 5:**
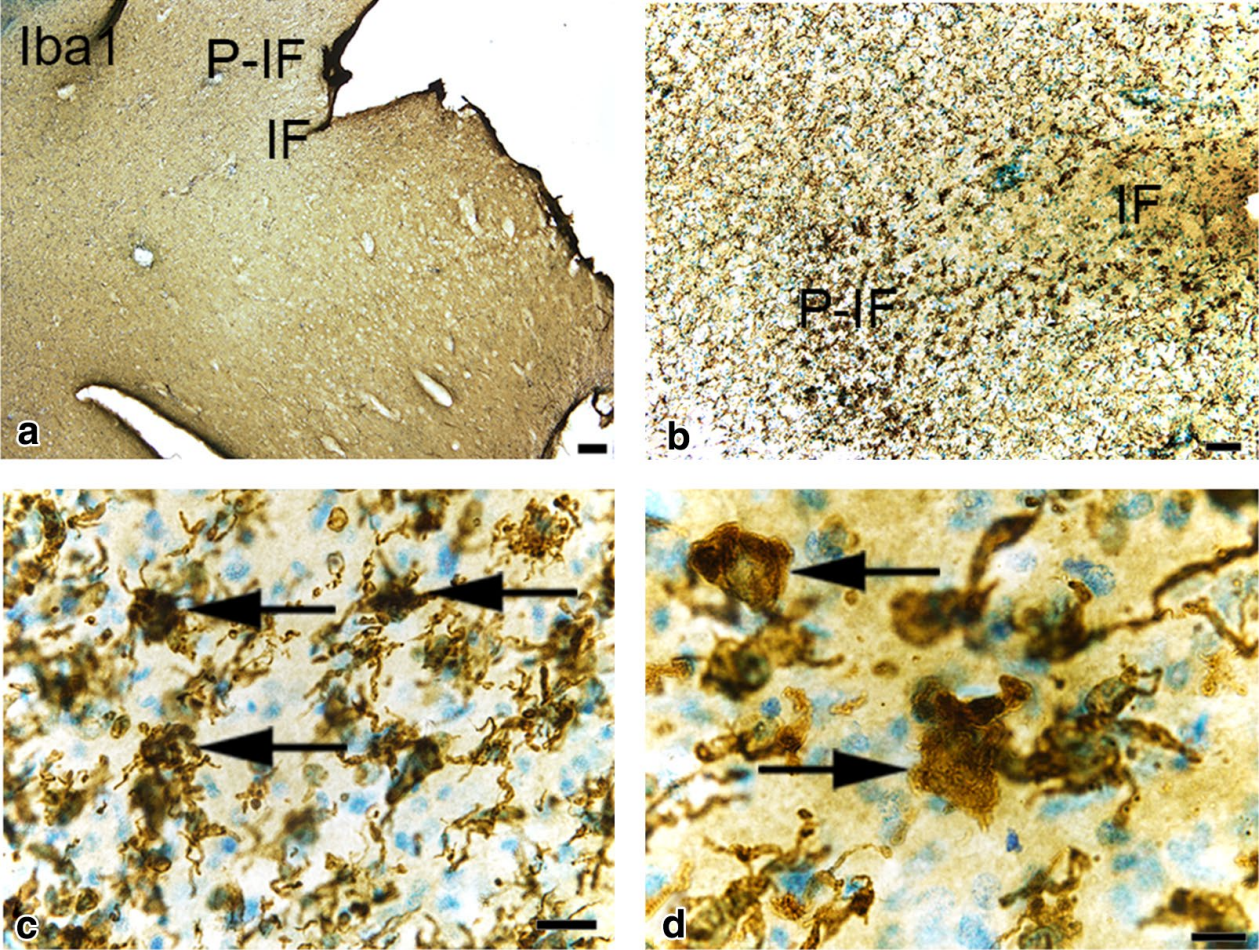
Photomicrographs showing microglial/macrophage activation in the cortical peri-infarct zone in the dog brain. *IF* infarct. *P*-*IF* peri-infarct area. Sections labelled for Iba1. *Arrows* reactive microglia. *Bars* **a** = 200 μm, **b** = 100 μm, **c** = 30 μm, **d** = 20 μm. Note reactive microgliosis in the peri-infarct area

**Fig. 6:**
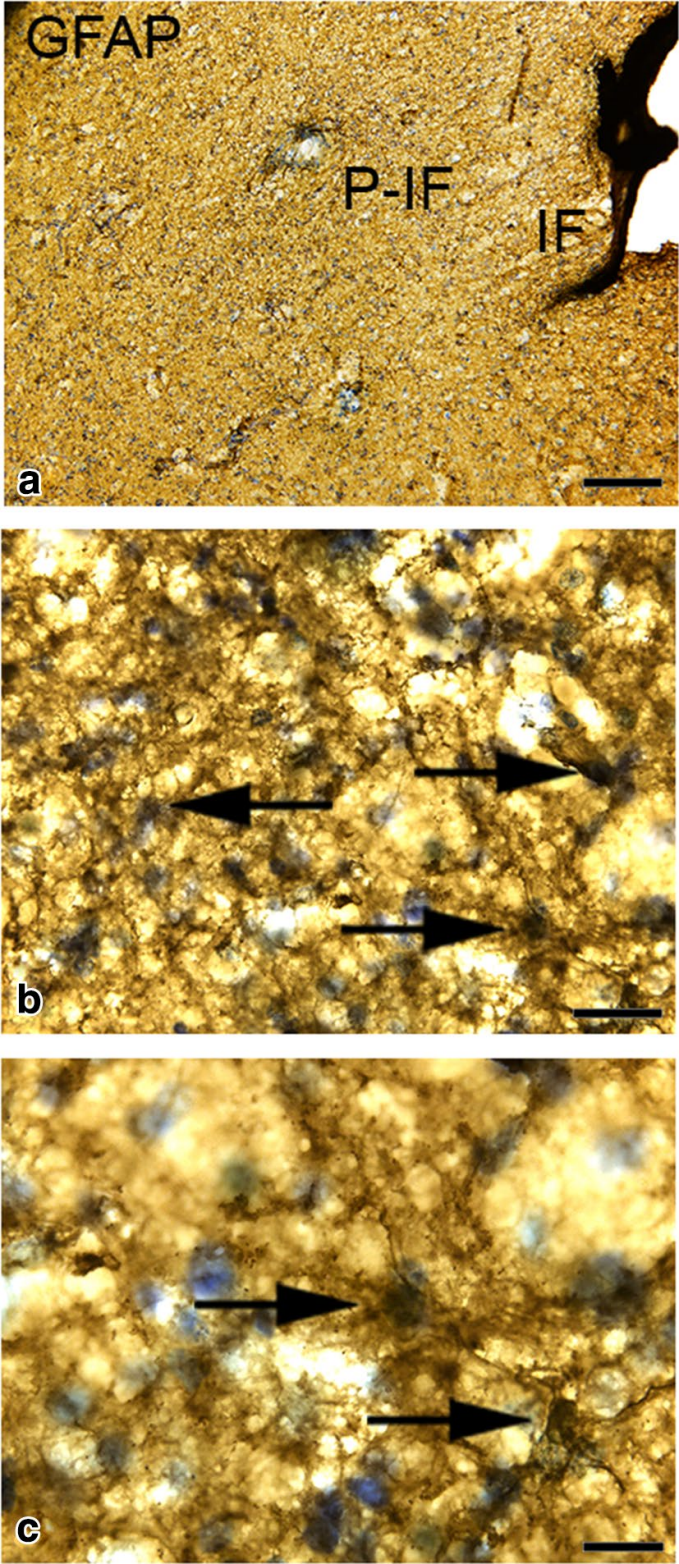
Photomicrographs showing astrocytosis in the cortical peri-infarct zone in the dog brain. *IF* infarct. *P*-*IF* peri-infarct area. Sections labelled with a GFAP antibody. *Bars* **a** = 200 μm, **b** = 30 μm, **c** = 20 μm. Note the reactive astrocytosis

Histologically, the brain had normal architecture and normal grey and white matter structure. There were no signs of atherosclerosis. In the infarcted areas of the right hemisphere, there was loss of neurons and glial cells and commencement of liquefactive necrosis. Neutrophil granulocyte and macrophage infiltrations were present within the necrotic areas of the parenchyma in accordance with the three-day post lesion time frame (Fig. 3). Ischaemic neuronal injury (shrunken cell somas and pyknotic nuclei) and loss of neurons were significantly more widespread than suggested by the size of the gross lesion (Fig. 4). Further morphological analysis of ischaemic neurons was limited due to the thickness and fragmentation of the vibratome sections. Immunohistochemical labeling for Iba1 revealed evident microglial/macrophage reactivity around the ischaemic lesion (Fig. 5). Numerous round macrophage-like cells (subsequently referred to as ‘macrophages’) had accumulated at the margin of the lesion as well as in the adjacent degenerated ischaemic parenchyma. Microglia displayed reactive microgliosis with various morphologies including the reactive macrophage phenotype in the vicinity of the lesion. Rod-shaped microglia were identified as well. A gradient of microglial reactivity was observed in the peri-infarct zone commencing with lightly reactive microglia far from the lesion (Fig. 5). Closer to the infarct, microglia appeared more reactive with hypertrophy and hyperramification of processes and a bushy appearance. In the contralateral hemisphere there was a circumscribed area of cortical microglial activation in the cerebral cortex demonstrating anterograde axonal (Wallerian) degeneration of commissural fibres from the right hemisphere cerebral cortex. Reactive astrocytosis was observed in the peri-infarct and characterised by increased GFAP expression (Fig. 6).

The frail texture of the infarcted areas of the right hemisphere resulted in fragmentation of the tissue when sectioned by the vibratome. The numerous fragments from each section made the exact anatomical location of the pathological changes within each section difficult to discern.

## Conclusions

Neuropathological changes in the affected area of the dog brain corresponded well to what has previously been described for 3-day-old infarcts in humans [33, 34] and experimental murine models [35], and included the presence of injured neurons, reactive microgliosis and astrocytosis as well as neutrophil granulocyte and macrophage infiltrations in the peri-infarct area.

In the canine ischaemic stroke brain, reactive microglia were found in the peri-infarct. The pathological changes observed in the present study are similar to those in the murine permanent MCA occlusion experimental model [36, 37]. Microglia are known to monitor the microenvironment of the brain and to react instantly to injury by undergoing morphological and functional changes [37, 38], thus neuronal death is suspected to induce transformation to phagocytic microglia with ameboid morphology as observed closest to and in the necrotic tissue in the present study [39]. Following the dynamic role of microglia in relation to the formation of the ischaemic lesion, microglia are the subject of a growing research interest [40, 41].

Astrocytosis was demonstrated in the cortical peri-infarct zone. Astrocytes function among other by maintaining the vascular tone changes following neuronal activity, and are capable of both secreting and absorbing neural transmitters. Immediately following injury to the brain, reactive astrocytosis develops. While a negative effect of astrocytosis by increasing infarct size has been shown [42], astrocytes at the same time have the potential to decrease the detrimental excitotoxicity [43, 44]. It is further known, that astrocytes in damaged tissue can induce a microglial response [37]. Whether astrocytes are primarily beneficial in terms of recovery or only exacerbate lesion progression is thus controversial [45]. Accordingly, this cell type should be further studied in animal models of ischaemic stroke, including the dog.

Neutrophil granulocytes were recognized based on nuclear morphology, which is a method that has previously proved reliable when evaluating TB stained sections [46]. In the present study, infiltration of neutrophil granulocytes into the necrotic centre of the canine brain parenchyma was observed (Fig. 3). This is in accordance with previous reports from experimental studies in rats and mice, which have shown that neutrophil migration into the parenchyma of a brain affected by ischaemic stroke peaks within the first 48 h [47, 48]. However, neutrophilic reactions following ischaemic stroke are not fully understood [49–52]. In humans, neutrophilic granulocytes are known to play a potentially harmful role with regard to infarct progression [53, 54]. Consequently, neutrophils in ischaemic stroke have been studied with the aim of developing novel treatments. Investigated potential targets include inhibiting activation, recruitment, and transmigration of neutrophilic granulocytes [49]. In humans, the proportion of leukocytes made up of neutrophils in the peripheral blood is approximately 50–70% [55]. In contrast, neutrophils in mice only constitute around 8–24% of the peripheral blood leukocytes [56], while the dog, interestingly, has a peripheral blood composition highly similar to humans with neutrophils forming approximately 60–80% of the peripheral blood leukocytes [57]. It would therefore be of interest to investigate the relationship between neutrophils and blood–brain barrier breakdown, haemorrhagic transformation, and the impact on final neurological outcome [49] in dogs with spontaneous ischaemic stroke.

When evaluating the dog as a potential spontaneous animal stroke model, it seems relevant whether the ischaemic stroke was caused by a local thrombus or by an embolus. In the present study, a thrombus or embolus was neither identified at necropsy nor at histological examination even though this was the suspected underlying cause. This might, however, be explained by the fact that embolus reduction in vivo as well as post-mortem in dogs usually takes place within a few hours [58]. In the present case, however, an embolus as the underlying cause of the infarct was strongly suspected due to the presence of petechial haemorrhages indicating haemorrhagic transformation, which is typically seen with embolic infarcts in humans [59]. In humans, the majority of ischaemic stroke events are caused by thromboembolism [55]. Atherosclerosis, which is the most frequent type of vascular pathology associated with arterial thrombosis in humans, seems rare in dogs and is most often associated with diabetes mellitus or hypothyroidism [16, 58]. Even though the T4 and free T4 levels were low and TSH was increased in the dog reported here, there were no clinical signs of concurrent hypothyroidism and no atherosclerosis was identified on histopathology. This further support the hypothesis of an embolus having caused the ischaemic stroke in the dog investigated.

The most common subtype of ischaemic stroke in humans is MCA territory infarcts [60], and the majority of animal models therefore aim at mimicking this subtype [61]. MCA occlusion is also a common subtype of spontaneous stroke in dogs [2], and thus offers an interesting spontaneous animal stroke model. So far, experimental studies have provided a substantial insight into the pathophysiology of ischaemic stroke, but effective neuroprotective drugs in experimental studies have failed when tested in human patients. The translational gap may, in part, be a result of the animal models not being able to mimic the complexity of the human disease appropriately [62]. A benefit of studying the pathophysiology of spontaneous stroke in dogs is that confounding factors such as anesthesia and surgical trauma of experimental models are avoided. Further, the similarities between the basic neuroanatomy of the canine and the human brain might explain the resemblance between the clinical disease observed in dogs and in humans with regard to associated neurological deficits and final outcome [2].

Ischaemic stroke seems to be less common in dogs than in humans [63]. The reasons for this remain unclear, but possible explanations could be the presence of vascular anastomoses in the canine brain, the rare occurrence of atherosclerosis in dogs [64] and the rapid dissolution of clots in dogs [58]. The low incidence of ischaemic stroke in dogs poses a hindrance to a widespread use of the dog as a spontaneous animal model for human ischaemic stroke. However, as studies regarding drug development for ethical reasons cannot be carried out in dogs, dogs could never fully replace existing animal stroke models. Instead, important investigations of the pathophysiology of spontaneous ischaemic stroke in dogs may contribute to bridge the translational gap between human patients and experimental animal models.

Our results are based on investigations of a single dog brain and thus cannot stand alone. In future, they should be followed by larger comparative studies, preferably using a multicenter design, which can ensure a high number of brains and support evidence-based conclusions. It would be of interest to perform further neuropathological characterisation of the reactions of neurons and neuroglia at different post stroke time points and investigations of vascular pathology seem highly relevant. Furthermore, white matter neuropathology has previously been linked to clinical deficits in humans with ischaemic stroke [65]. It would therefore also be of interest to investigate such white matter lesions in dogs.

## Abbreviations

DAB: 3,3′-diaminobenzidine
FCS: foetal calf serum
GFAP: glial fibrillary acidic protein
HE: haematoxylin and eosin
IHC: immunohistochemical
LFB: luxol fast blue
MCA: middle cerebral artery
MRI: magnetic resonance imaging
PBS: phosphate-buffered saline
T4: thyroxine
TB: toluidine blue
TBS: tris-buffered saline
TEG: tris-EGTA buffer
TSH: thyroid stimulating hormone
